# The Clinical Experiences of Urine Metabolomics of Genitourinary Urothelial Cancer in a Tertiary Hospital in Taiwan

**DOI:** 10.3389/fonc.2021.680910

**Published:** 2021-07-30

**Authors:** Horng-Heng Juang, Shao-Ming Chen, Gigin Lin, Meng-Han Chiang, Chen-Pang Hou, Yu-Hsiang Lin, Pei-Shan Yang, Phei-Lang Chang, Chien-lun Chen, Kuo-Yen Lin, Ke-Hung Tsui

**Affiliations:** ^1^Department of Urology, Chang Gung Memorial Hospital at Linkou, Taoyuan, Taiwan; ^2^Department of Anatomy, School of Medicine, Chang Gung University, Taoyuan, Taiwan; ^3^Department of Urology, Taipei City Hospital, Heping Campus, Taipei, Taiwan; ^4^Department of Medical Imaging and Intervention, Chang Gung Memorial Hospital, Taoyuan, Taiwan; ^5^Imaging Core Laboratory, Institute for Radiological Research, Chang Gung Memorial Hospital and Chang Gung University, Taoyuan, Taiwan; ^6^Graduate Institute of Clinical Medical Sciences, College of Medicine, Chang Gung University, Taoyuan, Taiwan; ^7^Department of Urology, Shuang Ho Hospital, Taipei Medical University, Taipei, Taiwan

**Keywords:** genitourinary urothelial cancer, urine metabolomics, bladder, urothelium, neoplasia

## Abstract

Few studies have addressed the impact of diagnostic urine metabolites and the clinical outcomes associated with genitourinary urothelial (GU) cancer to date. Furthermore, longitudinal analysis of the dynamics of urine metabolites contributing to the detection of GU cancer has not yet been fully investigated; therefore, the discovery of novel diagnostic urine biomarkers is of enormous interest. We explored the correlation of the urine metabolomic profiles to GU cancers. The aqueous metabolites of the GU cancer and the control were also identified and analyzed through high-resolution1H nuclear magnetic resonance (NMR) spectroscopy. Compared with the control, the urine metabolites of the tumor were studied in relation to changes over time in a linear mixed model for repeated measures. The urine metabolites of sixty-three (44 male and 19 female) patients with GU cancers were systemically analyzed. The urine metabolite profile in GU cancer was significantly higher than those in the control group (p<0.05). Sevenurine metabolites including histidine, propylene glycol, valine, leucine, acetylsalicylate, glycine, and isoleucine as well as other pathways were identified statistically and were significantly associated with GU cancer detection with longitudinal analysis. We discovered that histidine, propylene glycol, valine, leucine, acetylsalicylate, glycine, isoleucine, succinic acid, lysine2-aminobutyric acid, and acetic acid are involved significantly in all types of male patients in whom the type (upper tract) of urine metabolites were found to be statistically significant compared with the control. We did not find any statistical significance in urine biomarkers between female and male patients. However, a statistically insignificant correlation was found among the grade and stage with the metabolites.

## Introduction

Genitourinary urothelial (GU) cancer is one of the common cancers in Taiwan. In 2012, urologic cancer accounted for 10.0% of all the new malignant cases. In Taiwan, the incidence of upper tract urothelial carcinoma with chronic kidney disease accounts for 23.4% (1). However, one to two new cases per 100,000 inhabitants are found in Western countries. Bladder cancer is the ninth most common cancer among men according to the Taiwan Cancer Registry Annual Report for 2014 (2, 3). Painless hematuria, either microscopic or macroscopic, is the most common symptom, prompting the urologist into performing further studies. At times, the tedious process of arranging and undergoing examinations could cause a delay in diagnosis and treatment. Even after the complete resection in cases of localized disease, GU cancer harbors a significant risk for recurrence and metastatic spread. Tumor markers are the specific samplings to diagnose malignancy and monitor disease progression. Different cancers have different tumor markers for the differential diagnosing of visceral malignancy; such as prostate specific antigen (PSA) for prostate cancer (4, 5), carcinoembryonic antigen (CEA) for colon cancer (6), and vanillylmandelic acid (VMA) for pheochromocytoma (7). Regardless of the different degrees of specificity and sensitivity, tumor markers give clues to clinicians to the malignant potential. Compared with those from healthy subjects, serum samples from bladder cancer (BC) patients show decreased levels of isoleucine/leucine, tyrosine, lactate, glycine, citrate, as well as increased levels of lipids and glucose. The results reveal the disturbed metabolic pathways of aromatic amino acids, glycolysis, and citrate cycle, as well as lipogenesis metabolism in BC patients (8).

Owing to the characteristics of epithelial cancer of the GU tract, tumor markers are not easily established for differential diagnosis. It is our team’s effort to find any urinary metabolomics to lineate the malignant potential of the GU cancer. Metabolomics may offer practical solutions to traditional diagnostic methods for GU cancer (9–11). Urine metabolomics is a promising approach for urothelial cancer detection and marker discovery since, (1) urine is in direct contact with urothelial cells; (2) the metabolites released from the urothelial tract and bladder cancer cells may be enriched in urine samples. Metabolic profiles from urine provide a fingerprint for each individual, containing a significant amount of information on age, gender, lifestyle, dietary intake, and disease history. Metabolites are not merely the end product of gene expression; they are also the result of the interaction of the system’s genome with its environment.

Liquid chromatography-mass spectrometry (LC-MS) and High-resolution1H nuclear magnetic resonance (NMR) spectroscopy are the common instruments for analyzing urine metabolomics (12, 13). The latter was used in our study to investigate the changes in urinary metabolites with respect to age. We hypothesized that changes in the metabolites present in the urine correlate with GU cancer stage/status.

High-resolution 1H nuclear magnetic resonance (NMR) spectroscopy is widely used to measure dynamic changes in metabolite concentrations (13). The objective of this prospective study was to analyze urine metabolites from the samples of patients aged 35 to 92 years of age. Changes in urinary metabolites with respect to type, grade, and stage were assessed, and the relationship with cancer development was examined.

## Materials and Methods

### Study Population

We screened a consecutive cohort of patients with GU cancer. From March 2011 to August 2019, we collected spot urine samples of enrolled subjects with GU cancer. The pathologists reviewed hematoxylin and eosin stain slides with suspected cancer. The inclusion criteria were (1) histologically confirmed transitional cell carcinoma of GU cancer, (2) surgical resection of primary GU cancer, and (3) complete pathological, surgical, treatment, and follow-up data. The control group included normal patients, benign diseases of angiolipoma, and simple renal cysts with hematoma. The number of control patients was thirteen (eight female and 5 male) patients with an average age of sixty-seven. The sample collections were started at the time of the diagnosis. Informed consent forms were signed by the patients in a tertiary referral center, with a dedicated GU cancer interdisciplinary team screening patient enrollment. The exclusion criteria were (1) patients who received neoadjuvant chemotherapy or chemoradiation therapy, (2) tumor size < 1 cm on computed tomography (CT), and (3) prior to genitourinary surgery. The study was approved by the ethics committee of Chang Gung Memorial Hospital.

### Sample Processing

All urine samples were collected in the morning and immediately stored at −80°C in aliquots until required. Urine samples were split into several aliquots and stored at -80°C immediately until analyzed. Briefly, 900 μL of thawed urine was mixed with 100 μL of 1.5 M phosphate buffer (pH 7.4) in deuterium water which contained 0.04% 3-(trimethylsilyl)-propionic-2,2,3,3-d4 acid sodium salt (TSP) as an internal chemical shift reference standard in deuterium water. The mixtures were vortexed for 20 s and centrifuged at 12,000g at 4°C for 30 min. A 600 μL of the supernatant was loaded to a standard 5 mm NMR tube for further analysis.

### NMR Acquisition

NMR experiments were performed at Chang Gung Healthy Aging Research Center, Taiwan. 1H-NMR spectra were acquired on a Bruker Avance 600 MHz spectrometer (Bruker-Biospin GmbH, Karlsruhe, Germany) equipped with a 5 mm CPTCI ^1^H cryoprobe. For each spectrum, 64 transients were collected into 64K data points using a spectral window of 20 ppm during the relaxation time of 4 s. Prior to Fourier transformation, all ^1^H-NMR spectra were processed with zero-filling and exponential line-broadenings of 0.3 Hz. The acquired spectra were manually phased, baseline corrected and calibrated the internal TSP signal to δ 0.0 ppm using TopSpin 3.2 software (BrukerBioSpin, Rheinstetten, Germany).

### NMR Data Processing

NMRProcFlow (https://www.nmrprocflow.org) is an open source software that provides comprehensive tools for processing and visualising 1D NMR data. The raw ^1^H-NMR spectra were imported into NMRProcFlow 1.2 for PPM calibration, baseline correction, alignment, spectra bucketing, and data normalization. ^1^H-NMR urine spectra were calibrated to creatinine signal (δ 4.05 ppm) and intelligent bucketed after baseline correction and alignment. Metabolite identification was performed by using Chenomx NMR Suite 8.0 professional software (Chenomx Inc., Edmonton AB, Canada). Metabolic alterations of GU tumors versus different gender, type, stage, and control were investigated. From the heat maps, we compared the type, stage, and grade. We found the upper tract of the tumor was correlated to the metabolite significantly. We could not see any significant change in stage and grade. Groups were compared by defining variable importance in projection score of > 1.2, a fold change value or its reciprocal of > 1.2, and a P value of < 0.05 as a significant difference.

### Data Processing and Statistical Analysis

The exported bucketing data of ^1^H-NMR spectra were uploaded to MetaboAnalyst 4.0 (http://www.metaboanalyst.ca) with mean-centered, generalized log transformation and scaled by Pareto scaling. To find out the importance of metabolites between different groups, partial least squares-discriminant analysis (PLS-DA), independent t test, and fold change were applied. The pathway analyzed potential biomarkers, for which the *p* value was smaller than 0.05, to identify the association among these biomarkers. The coordinate values of the observations on the plane are called scores, and hence the plotting of such a projected configuration is called a score plot.

The variable importance in the projection (VIP) score is a measure of a variable’s importance in the PLS-DA model. The variable importance in the projection (VIP) value of each variable in the model was calculated to indicate its contribution to the classification. A higher VIP value represented a stronger contribution to discrimination among the groups. VIP values > 1.2 were considered significant.The fold change is the ratio of two values. It measures how much variable has changed between the two measurements and summarizes the contribution a variable makes to the model.

A heatmap is a data visualization technique that shows the magnitude of a phenomenon as color in two dimensions. The color variation may be by hue or intensity, giving obvious visual clues to the reader about how the phenomenon is clustered or varies over space.

Data were compared by 2-sample or paired Student’s *t* test, analysis of variance, or chi-square test, when appropriate. The results are expressed as the mean ± standard deviation for continuous variables and as the number (percent) for categorical variables. A *P* value of < 0.05 was considered significant.

The false discovery rate (FDR) is the expected proportion of type I errors. A type I error is where you incorrectly reject the null hypothesis. In other words, we get a false positive.

## Results

### Patients’ Characteristics

Sixty-three patients with genitourinary urothelial cancer were reviewed, with a median age of 71years (range,35-92 years). Twentytwo patients belonged to upper tract urothelial cancer (UTUC), including1 4 male and 8 female patients. There were 41 patients with bladder cancer (lower tract UC), 30 were male, and 11 were female. We prospectively enrolled these patients in a continuous cohort. No statistically significant difference in demographics was observed between the two groups (**Table 1**).

**Table 1:** Characteristics of patients.

	Genitourinary Urothelial Cancer		
	Upper Tract (22)	Lower Tract (41)	P-value
Age (median yr, range)	71.0 years (range, 35-92 years)		
Mean (SD)	71.4 (11.9)	70.5 (15.4)	0.798
Median [Min, Max]	73.0 [35.5, 86.0]	70.8 [40.3 92.1]	
Sex (male/female)	14/8	30/11	0.618
Grading:			
High (male/female)	18 (11/7)	28 (20/8)	0.392
Low (male/female)	4 (3/1)	13 (10/3)	
Stage:			
Tis	1		
Ta	23		
T1	17		
T2	12		
T3	7		
T4	3		
Advanced	5 (22.7%)	6 (14.6%)	0.647
Local	17 (77.3%)	35 (85.4%)	

### Identification of Metabolites and Metabolic Pathways Between GU Cancer and Male Patients Among All Different Groupings of Cancer.

The female patients did not show any significant change in metabolic pathways and urine metabolites in all types of the tumor (**Table 2**). By using the VIP score and Fold Change of the metabolites, we compared the type, stage, and grade of cancer (**Table 3**). We found that histidine, propylene glycol, acetylsalicylate, glycine, valine, leucine, guanidoacetic acid, and 2-aminobutyric acid to be the most significant change in the type of the tumor (**Table 3**). The biochemical pathways involved contained aminoacyl-tRNA biosynthesis, valine, leucine, isoleucine biosynthesis, and degradation; nitrogen metabolism; cyanoamino acid metabolism (**Table 4**). They were found to be statistically significant in all types of tumors. On the other hand, we found four more pathways, namely pyruvate metabolism, propanoate; biotin metabolism, and lysine degradation except cyanoamino acid metabolism to be involved significantly in male patients with all types of tumor.

**Table 2:** Statistically insignificance of the metabolites in female patients.

Metabolites	Type			Type					
				Male (14 : 30)			Female (8 : 11)		
	VIP score	Fold Change	*p*	VIP score	Fold Change	*p*	VIP score	Fold Change	*p*
Histidine	2.33	0.38	0.001	2.11	0.34	0.002	1.41	0.38	0.121
Propylene glycol	2.29	0.09	0.007	2.05	0.24	0.011	1.39	0.03	0.235
Valine	1.52	0.24	0.020	1.35	0.21	0.026	1.20	0.24	0.171
Leucine	1.41	0.26	0.031	1.30	0.35	0.020	1.11	0.17	0.235
Acetylsalicylate	1.16	0.55	0.036	1.22	0.57	0.016	0.44	0.48	0.571
Glycine	1.23	0.38	0.045	1.36	0.51	0.013	0.53	0.24	0.540
2-Hydroxyisobutyric acid	1.02	0.49	0.050	0.64	0.76	0.128	1.25	0.26	0.108
Isoleucine	1.23	0.38	0.055	1.24	0.37	0.033	0.85	0.32	0.334
Succinic acid	0.92	0.64	0.065	1.06	0.64	0.014	0.56	0.55	0.411
Lysine	0.98	0.46	0.079	0.98	0.59	0.040	0.68	0.30	0.407
2-Aminobutyric acid	0.87	0.58	0.143	1.02	0.56	0.048	0.59	0.49	0.457
Acetic acid	0.53	1.03	0.334	0.96	0.64	0.036	0.07	1.27	0.927

Red texts are “statistically significance”.

**Table 3:** Comparison among different grouping (Type, Stage, Grade).

Metabolites	Type			Stage			Grade		
	VIP score	Fold Change	*P*	VIP score	Fold Change	*p*	VIP score	Fold Change	*p*
Histidine	2.33	0.38	**0.001**	1.41	0.64	0.237	0.04	1.17	0.812
Propylene glycol	2.29	0.09	**0.007**	1.09	0.15	0.451	0.33	1.82	0.544
Acetylsalicylate	1.16	0.55	**0.036**	0.52	0.81	0.572	0.79	0.87	0.329
Glycine	1.23	0.38	**0.045**	0.39	0.46	0.706	0.07	1.49	0.656
Valine	1.52	0.24	**0.020**	0.10	1.66	0.930	0.53	2.59	0.329
Leucine	1.41	0.26	**0.031**	0.85	0.71	0.434	0.13	2.83	0.616
Guanidoacetic acid	0.28	1.19	0.591	0.18	0.97	0.840	1.08	1.75	**0.037**
2-Aminobutyric acid	0.87	0.58	0.143	1.12	1.02	0.254	1.37	2.07	**0.019**

Red texts are “statistically significance”.

**Table 4:** Statistically significance of the metabolites in the type of male patients in different pathway analyses.

	Metabolites	Pathway Name	Match Status	p	FDR	Impact
Type_male	Histidine	Lysine degradation	2/39	0.013	0.171	0.000
	Propylene glycol	Biotin metabolism	2/47	0.018	0.210	0.147
	Valine					
	Leucine	Biotin metabolism	1/11	0.049	0.492	0.000
	Acetylsalicylate	Pyruvate metabolism	2/32	0.009	0.167	0.106
	Glycine					
	Isoleucine	Propanoate metabolism	2/35	0.010	0.167	0.001
	Succinic acid	Nitrogen metabolism	2/39	0.013	0.171	0.000
	Lysine					
	2-Aminobutyric acid	Lysine degradation	2/47	0.018	0.210	0.147
	Acetic acid	Biotin metabolism	1/11	0.049	0.492	0.000

The types of the tumor were compared by defining variable importance in a projection score of > 1.2, a fold change value or its reciprocal of > 1.2, and a *p* value of < 0.05 as a significant difference. We discovered that histidine, propylene glycol, valine, leucine, acetylsalicylate, glycine, isoleucine, succinic acid, lysine, 2-aminobutyric acid, and acetic acid to be involved significantly in all types of male patients.

### Metabolite-Concentration Distribution of GU Cancer Related to Its Type Using the PLS-DA

**Figure 1:**
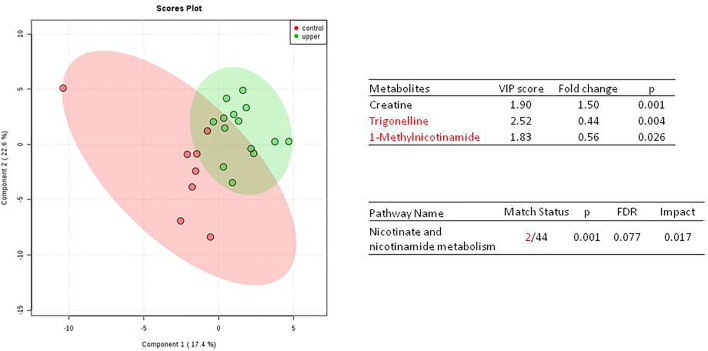
Metabolite-concentration distribution of GU tumor and control using the PLS-DA.The tumor (green) *vs* healthy control sample (red) were analyzed; the metabolites of trigonelline and 1-methylnicotinamide were significantly increased in the GU cancer group. (p < 0.001).

**Figure 2:**
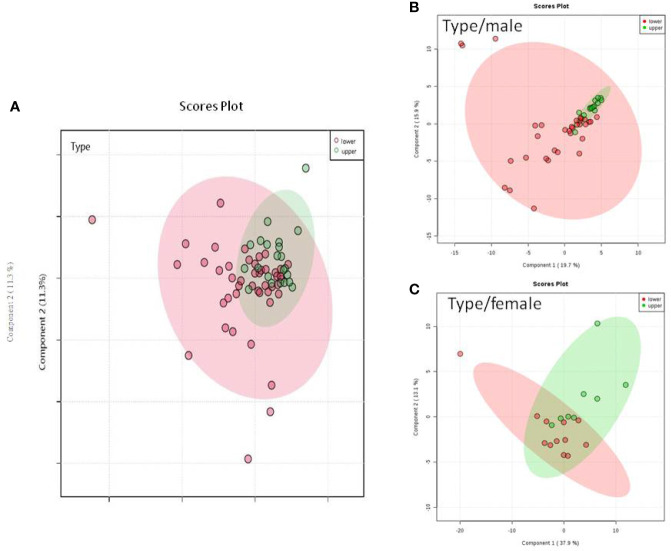
Metabolite-concentration distribution GU cancer and its type using the PLS-DA. **(A) ** Denotes the upper and lower parts of GU tumor. **(B)** The different type of tumor related to male. **(C)** The different types of tumor related to female.

**Figure 3:**
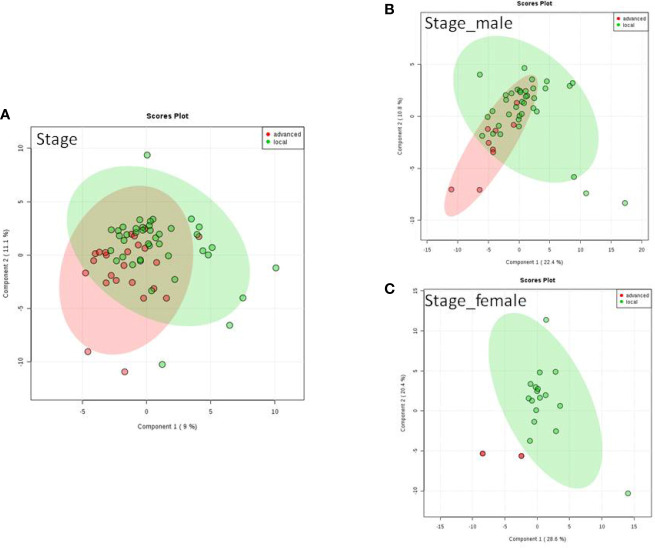
Metabolite-concentration distribution of GU cancer and its stage usi ng th e PLS-DA. **(A)** Denotes in staging of advanced and local cancer. **(B)** Denotes in staging of advanced and local in male. **(C)** Denotes the staging of advanced and local cancer in female.

**Figure 4:**
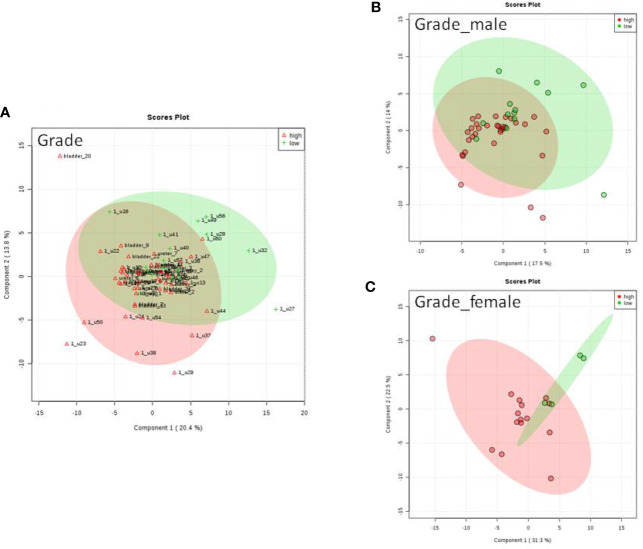
Metabolite-concentration distribution GU cancer and its grading using the PLS-DA. **(A)** Denotes the grading of cancer. **(B)** Denotes the grading of cancer in male. **(C)** Denotes the grading of cancer in female.

We do found that the metabolites were significantly changed in the upper tract of the GU tumor compared with the control group (**Figure 1**). The pathways of the nicotinate and nicotinamide metabolism were assessed. We found that the levels of creatine; trigonelline and 1-methylnicotinamide significantly increased in the type of upper part in the male patients. However, neither type (upper tract UC and lower tract UC) nor gender related to the type of the tumor were found to have a significant change in urine metabolite (**Figure 2**). On the other hand, we did not see any significant change in tumor staging (Ta-T1 versus T2-T3) (**Figure 3**) and tumor grading (**Figure 4**) associated with urine metabolite compared with a different gender.

### Distribution of Urine Metabolites in the Heatmap

**Figure 5:**
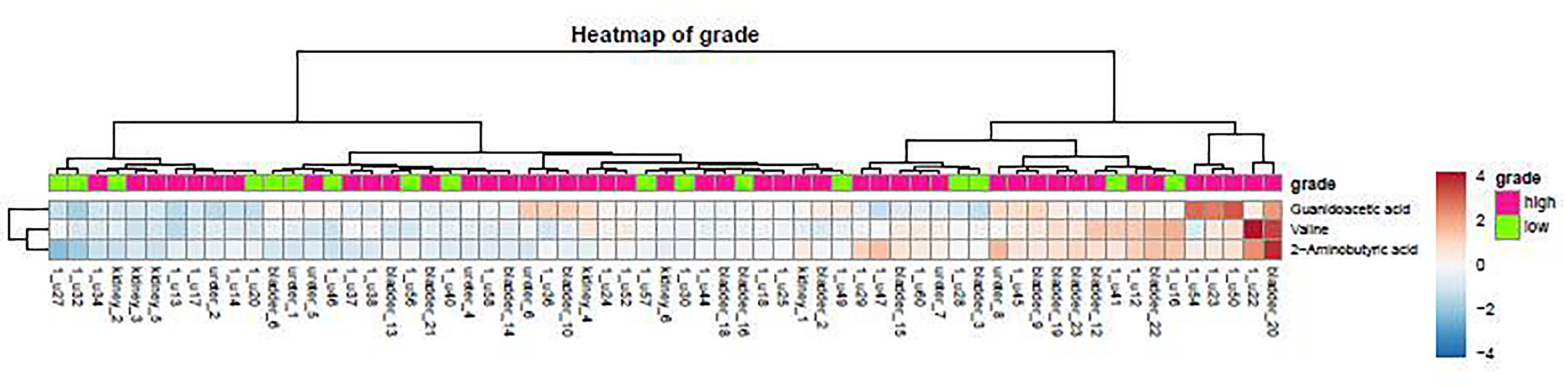
The distribution of urine metabolites of the tumor grade was observed by using the heatmap. The metabolite is not much difference between high tumor grading of UC (red) and low tumor grading (green).

**Figure 6:**
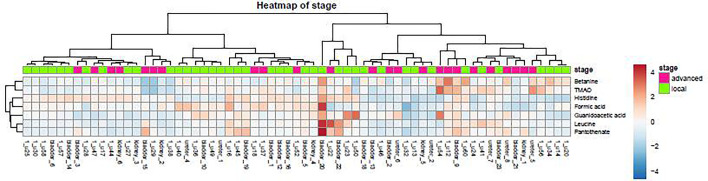
The distribution of urine metabolites of the tumor stage was observed by heatmap. The metabolite is not much different between advanced UC (T2-T3, red) and local tumor stage (Ta-T1, green).

**Figure 7:**
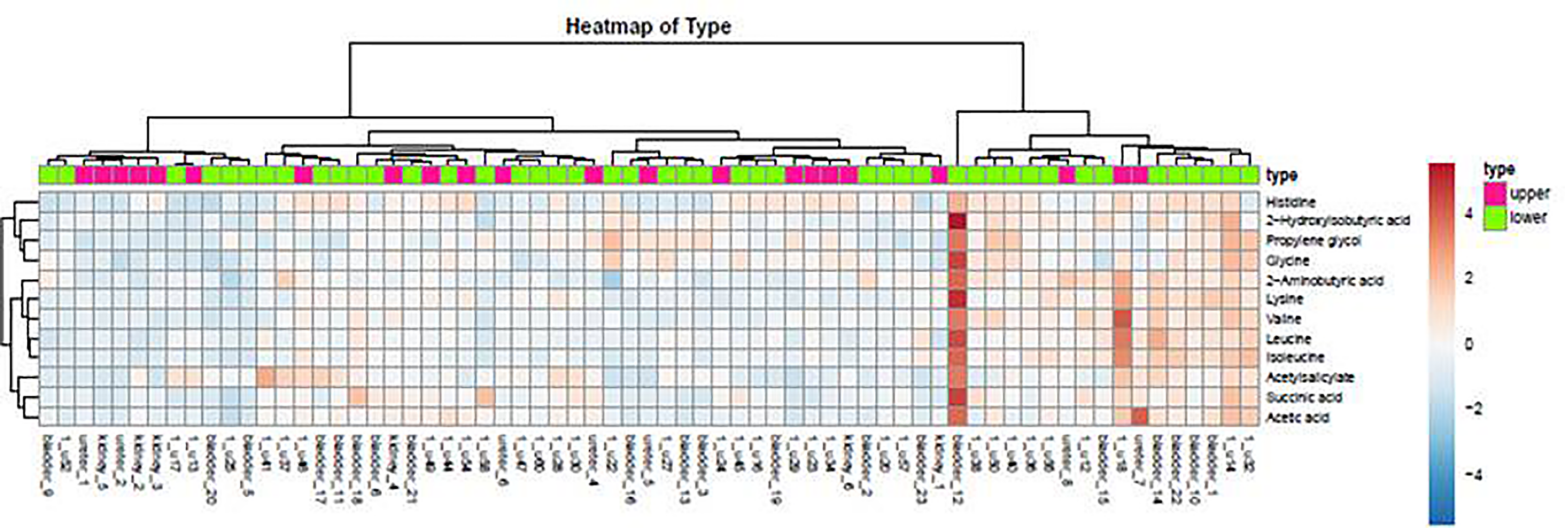
The distribution of urine metabolites of the type of UC was observed by heatmap. The metabolites showed a significant difference between upper tract GU cancer (red) versus lower tract GU cancer (green).

As demonstrated in the heatmap of **Figure 5**, red and green denote the high grade and low grade tumors, respectively. The clustering of sensitive metabolites, namely guanidoacetic acid and 2-Aminobutyric acid were significantly segregated in the high grade of the tumor. From this model, a few metabolites with high variable importance projection (VIP) scores and fold change were selected. By contrast, there was no significant change in the non-sensitive urine metabolites in either advanced (red) or local (green) stage tumors (**Figure 6**). We found sensitive urine metabolites, which were selected from VIP scores and fold change, and which were significantly associated with the type of tumor, namely histidine, proplylene glycol, acetysalicylate, glycine, valine, leucine, 2-aminobutyric acid, 2-hydroisobutyric acid, isoleucine, succinic acid, lysine and acetic acid (**Figure 7**). They are almost exclusively associated with the more localized type (green) of tumor.

## Discussion

Genitourothelial cancer may not be one of the most common carcinomas in the world, but it is, however, detrimental to a patient’s well-being. The metabolite distributions in tumor and healthy tissue could be clearly distinguished in GU cancer. A critical clinical implication of our GU cancer in metabolomics study is biomarker discovery. Urine metabolomics is a promising approach to urothelial cancer detection and biomarker discovery since urine is in direct contact with bladder epithelial cells and metabolites released from bladder cancer cells may be enriched in urine samples (14, 15).

Painless gross/microscopic hematuria is usually the first sign of urothelial cancer, however, the time to diagnosis could be late in stage. It was our goal to detect tumor markers in early cancer stage to lineate the prognosis of cancer. Metabolicprofiling provides a new approach to exploring the metabolic effects of many conditions in complex biological systems (16).

To our knowledge, the present study is the first report combining GU cancer with metabolomics data in Taiwan. Amino acids have been shown to contribute to various antioxidants and immunological activities relevant to cancerpathogenesis (12, 17). Quinolinate is associated with various disease conditions, such as infection and neurodegenerative disease. Another key significantly altered metabolite, α-ketoglutarate (also known as 2-oxoglutarate), is also within the “alanine, aspartate, and glutamate metabolism” pathway. This metabolite is the entry point to the citric acid cycle, which is increased in glycolysis, and is again consistent with the Warburg effect if it is a result of increased production by the tumor.

Using urinary metabolomics, our findings provide a link between the type of tumor and metabolites. Amino acids have been shown to contribute to various antioxidants in different cancers. In the cross-sectional analysis of this study, amino acids were identified to be significantly associated with cancer. Histidine, propylene glycol, valine, leucine, acetylsalicylate, glycine, and 2-hydroxyisobutyric acid are significantly associated with the type of cancer. This also indicates that clinical staging and grading of GU cancer should be monitored with a non-metabolite marker, although there are few metabolites significantly associated with grading. Furthermore, we were able to trace the different metabolic pathways back using the urine metabolites we found. However, clinical pathology is not significantly related to the metabolic pathway. On the other hand, a significant elevation in the concentration of taurine was observed in the urine of bladder cancer patients, which was below the sensitivity limit of 400MHz in control cases (16). Furthermore, A 32-metabolite/resonance signature descriptive of RCC was unveiled, successfully distinguishing RCC patients from controls in principal component analysis. This work demonstrates the value of a systematic metabolomics workflow for the identification of the robust urinary metabolic biomarkers of RCC (18).

To the best of our knowledge, this is a pioneer cohort study looking at a wide range of urine biomarker measurements in genitourinary urothelial cancer in a human model. In our preliminary report, urinary metabolomics is one of the useful biomarkers to make a differential diagnosis of different types of genitourinary urothelial cancer. This study provides an overview of metabolic changes in GU cancer which potentially provides information on the diagnosis and monitoring of genital urinary cancer. This study found that histidine, propylene glycol, valine, leucine, acetylsalicylate, glycine, and isoleucine arethemetabolics are potentially specific biomarkers for genitourinary urothelial cancer. Pathway analyses show taurine, alanine, aspartate, glutamate, and phenylalanine perturbed metabolism associated with non-muscle invasive bladder cancer. These results highlight the potential of ^1^H NMR metabolomics to detect bladder cancer (BC) recurrences through a non-invasive approach (18). Six highly sensitive biomarker candidates (urea, choline, methylguanidine, citrate, acetone, and β-hydroxybutyrate) were identified through an NMR-Based metabolomics study of canine bladder cancer (19). On the other hand, urine metabolomics using high performance liquid chromatography-mass spectrometry has the potential to become a noninvasive early detection test for bladder cancer (20, 21), but the screening test is less specific to urine biomarkers. In current practice, screening for GU cancer is not used because of the lack of an appropriate, cost-effective, and acceptable test that reduces morbidity and mortality. Therefore, larger studies are required to understand the role of these metabolites in GU tumors in greater detail and to validate this biomarker’s utility followed by its translation into a clinical setting in NMR-based urine metabolomic analysis. The metabolomic profiles of GU tumors and the control are distinct, and the type of tumor status is associated with downstream metabolic alterations in the genitourinary tract.

The small sample size and relatively low sensitivity of NMR-based metabolomic analysis of this study mean that it has limited statistical power. Given the small sample size and multiple comparisons, interpretation of the results must be cautious, even with validation data. In a future perspective, we would like to collect more patient survival data to understand what metabolites can affect cancer progression or patient prognosis. These more specific and sensitive markers enable diagnosis and could potentially serve as specific biomarkers for GU cancer. One important question remains: how long before GU cancer diagnosis does the urine level of various markers begin to rise above background levels as a tumor grows?

## Conclusions

Seven metabolites (histidine, propylene glycol, valine, leucine, acetylsalicylate, glycine, and isoleucine) were associated with the detection of the tumor. On the other hand, isoleucine, succinic acid, lysine, 2-aminobutyric acid, and acetic acid were significantly involved in all tumor types analyzed for male patients. These initial metabolomics studies provided urine biomarkers that can, more or less, differentiate GU cancer patients from healthy controls. This initial experiment found that the urine biomarkers are significantly related to the type of tumor. This pioneering study on urine metabolomics profiling based on GU cancer status has far reaching effect on urine biomarker discovery, elucidation of biochemical pathways, and novel diagnostic development. Urine metabolomics is a non-invasive screening test, and we hope this study will inspire more scientists to investigate urine biomarkers, regardless of their field.

## Data Availability Statement

The original contributions presented in the study are publicly available. This data can be found here: https://www.ebi.ac.uk/metabolights/, MTBLS2824.

## Ethics Statement

The studies involving human participants were reviewed and approved by ethics committee of Chang Gung Memorial Hospital. The patients/participants provided their written informed consent to participate in this study.

## Author Contributions

K-HT, Y-HL , C-PH, H-HJ, M-HC, C-lC, P-SY, and P-LC undertook conception and design. S-MC, GL, M-HC, and C-PH performed enrollment of patients and acquisition of data. K-HT, Y-HL, M-HC, C-lC, and P-LC drafted the manuscript. C-PH, Y-HL, M-HC, and H-HJ undertook the statistical analysis. C-lC and H-HJ performed analysis and interpretation of data. K-HT, Y-HL, C-PH, P-SY, and C-lC undertook supervision. All authors contributed to the article and approved the submitted version.

## Conflict of Interest

The authors declare that the research was conducted in the absence of any commercial or financial relationships that could be construed as a potential conflict of interest.
